# Profiling household double and triple burden of malnutrition in sub-Saharan Africa: prevalence and influencing household factors

**DOI:** 10.1017/S1368980021001750

**Published:** 2022-06

**Authors:** Aaron K Christian, Fidelia AA Dake

**Affiliations:** Regional Institute for Population Studies, University of Ghana, LG 96 Legon, Accra, Ghana

**Keywords:** Malnutrition, Anaemia, Household, Double burden, Triple burden, Sub-Saharan Africa

## Abstract

**Objective:**

Undernutrition and anaemia (the commonest micronutrient deficiency), continue to remain prevalent and persistent in sub-Saharan Africa (SSA) alongside a rising prevalence of overweight and obesity. However, there has been little research on the co-existence of all three conditions in the same household in recent years. The current study examines the co-existence and correlates of the different conditions of household burden of malnutrition in the same household across SSA.

**Setting:**

The study involved twenty-three countries across SSA who conducted Demographic and Health Surveys between 2008 and 2017.

**Participants:**

The analytical sample includes 145 020 households with valid data on the nutritional status of women and children pairs (i.e. women of reproductive age; 15–49 years and children under 5 years).

**Design:**

Logistic regression analyses were used to determine household correlates of household burden of malnutrition.

**Results:**

Anaemia was the most common form of household burden of malnutrition, affecting about seven out of ten households. Double and triple burden of malnutrition, though less common, was also found to be present in 8 and 5 % of the households, respectively. The age of the household head, location of the household, access to improved toilet facilities and household wealth status were found to be associated with various conditions of household burden of malnutrition.

**Conclusions:**

The findings of the current study reveal that both double and triple burden of malnutrition is of public health concern in SSA, thus nutrition and health interventions in SSA must not be skewed towards addressing undernutrition only but also address overweight/obesity and anaemia.

The 2020 Lancet series on the ‘dynamics of the double burden of malnutrition and the changing nutrition reality’ suggests that the global health community has been slow in acknowledging and responding to the high prevalence of the double burden of malnutrition, particularly in low and middle countries^(1)^. Historically, malnutrition has been linked with different population sub-groups, and the divide has mostly been by socio-economic status; undernutrition was linked to resource poor populations, while overweight and obesity were associated with the affluent^(2)^. This predisposition often led to a functional divide when addressing malnutrition challenges. However, over the past two decades, there have been indications that undernutrition and overweight/obesity are increasingly co-occurring and becoming a public health problem^(3)^ with no socio-demographic and or wealth/income groupings spared^(4)^. Additionally, although globally, no regional block has been exempted from the rising prevalence of overweight/obesity which has translated into the current obesity pandemic^(5)^, sub-Saharan Africa (SSA) has been observed to be experiencing a rapid rise in overweight/obesity, and the region also continues to be the only region where undernutrition is on the rise in almost all its sub-regions^(6)^. The rise in the burden of overweight and obesity in low-resource countries was highlighted by Barry Popkin in his seminal work on the nutrition transition, in which he projected that the rate of dietary changes such as consumption of energy-dense foods and increasing decline in physical activity among the poor could result in an upsurge of overweight and obesity in developing countries^(7,8)^.

These changes in lifestyle habits could also be viewed as socially constructed choices that are modulated or influenced by various commercial interests^(9,10)^. Furthermore, the commercial determinants of health, defined as ‘strategies and approaches used by the private sector to promote products and choices that are detrimental to health’, are now viewed as a critical element in understanding ways of addressing the current levels of malnutrition^(11)^. Additionally, the increasing penetration and intense advertising of sugar-sweetened beverages by both international and local food companies have a significant influence on the intake of sugars and thus contribute to overweight and obesity. This trend has edged some researchers to advocate for increased attention to be given to both the commercial and social determinants of health^(11,12)^, especially as it relates to malnutrition, particularly overweight and obesity.

In addition to the aforementioned challenges, the SSA region is also the hardest hit with regard to the consequence of climate change. The increasing temperatures and extreme rainfall that characterise climate change are altering suitable conditions for farming. Additionally, given its impact on agricultural systems, climate change significantly slows down progress towards improving the food security of households in SSA^(13)^. The work of the Intergovernmental Panel on Climate Change provides adequate evidence about the link between climate change, food insecurity and undernutrition, particularly among vulnerable populations in sub-Saharan Africa, due to crop failures, reduced food production and increased foodborne diseases. The interaction between climate change and food security is complex and its impact on malnutrition is multifaceted. On the one hand, there is evidence that whereas severe food insecurity could lower obesity, on the other hand, mild to moderate food insecurity has been shown to be associated with obesity^(14)^. The dynamics of how this paradox impacts household burden of malnutrition needs to be researched.

Increasingly, undernutrition and overweight/obesity are being observed to occur in the same community, in the same household and among different members of the same household^(3,15)^. Malnutrition at the household level is often observed among children and women, but this does not mean men are not affected^(16,17)^. The pattern of malnutrition among children and women in SSA is changing from the previously observed undernutrition among women and children to undernutrition among children and overweight/obesity among women and even more recently to overweight/obesity among both women and children^(18)^. A more nuanced examination shows the existence of even more permutations of undernutrition and overweight/obesity among women and children in the same household.

Aside undernutrition and overweight/obesity, other nutritional disorders with equally grave public health implications include micronutrient deficiencies with iron-deficiency anaemia being the most widespread^(19)^. Currently, the prevalence of iron-deficiency anaemia is the highest among all nutritional deficiencies around the world^(20)^ and in developing countries^(21)^. Unfortunately, over the past decade, SSA has achieved the lowest progress with respect to reducing the prevalence of iron-deficiency^(22,23)^. Consequently, SSA is particularly vulnerable to the burden of all forms of malnutrition; undernutrition, overweight/obesity and micronutrient deficiency and even worse, their simultaneous co-existence. And regardless of the condition of malnutrition, the consequences are largely negative. Childhood undernutrition for instance is associated with increased risk of mortality and poor cognitive development^(24)^, while overweight/obesity is associated with increased incidence of chronic non-communicable diseases^(25)^. Maternal overweight/obesity on the other hand is linked with various adverse maternal as well as fetal outcomes^(26)^. Consequently, the co-existence of undernutrition, overweight/obesity and anaemia exacerbate the burden of ill-health and retards development among household members.

Household burden of malnutrition can be classified by the collective burden among individual members of the household. Double/dual burden of malnutrition (DBM) is considered as the co-existence of maternal overweight and obesity along with child undernutrition within the same household^(27,28)^, while triple burden of malnutrition (TBM) refers to the co-existence of overweight/obesity, undernutrition and micronutrient deficiency^(29,30)^ in the same household. It is also worth stating that, there could be the co-existence of overweight/obesity and micronutrient deficiency in the same individual and this could occur in either a child or the mother (adult woman). A child can also simultaneously be overweight/obese and stunted. This is also another form of individual-level double burden of malnutrition^(31)^.

Double and triple burden malnutrition households are becoming more common and rising rapidly in SSA partly due to the ongoing rapidly evolving nutrition transition^(3,32)^. Additionally, whereas structural and institutional changes such as unregulated marketing of cheap processed foods and sugar-sweetened beverages and the lack of physical activity and physical activity spaces are identified as critical factors driving overweight/obesity^(33,34)^, the region’s continued high levels of food insecurity, HIV prevalence and per capita income have been identified to be associated with undernutrition^(35)^. Furthermore, although the double and triple burden of malnutrition is often reported as a regional or global phenomenon, its expressions and impacts are experienced at the micro-level in households. Thus, exploring correlates of malnutrition, particularly the double and triple burden of malnutrition at the household level, is critical for both public health programmers and policy makers to guide the development and implementation of appropriate policy interventions.

Addressing the double and triple burden of malnutrition in SSA will require understanding the intricacies and characteristics of households that have been affected. To our knowledge, overweight/obesity, undernutrition and anaemia in woman–child pairs within the same household have not yet been explored using nationally representative data across SSA. But considering that SSA already has weak health infrastructure, the emergence of this nutritional paradox compromises the regions development even further. Furthermore, if target 2·2 of the Sustainable Development Goals^(36)^ which aims at reducing all forms of malnutrition is to be achieved, the household drivers of the burden of malnutrition need to be effectively understood. Considering the above-mentioned context, we use nationally representative data across SSA, to explore correlates of the different classifications of household malnutrition, i.e. undernutrition, overweight/obesity, anaemia (as an indicator of micronutrient deficiency) as well as their co-existence in the same household. Such analysis will contribute significantly to effective ways of addressing the co-existence of undernutrition, overweight/obesity and anaemia in households in SSA.

## Materials and methods

### Data sources and procedures

The current study used data from the most recent Demographic and Health Survey (DHS) conducted across SSA where data were available. The DHS are standard nationally representative cross-sectional household-based surveys that are conducted approximately every 5 years. Data from the DHS provide indicators on population and health for multi-country comparison. The DHS programme started in the 1980s and has contributed to advancing global knowledge on health and population trends in low- and middle-income countries. All DHS are conducted using a standardised survey design and data collection procedures across participating countries. Typically, the DHS uses a stratified cluster sampling technique to select census enumeration areas based on probability proportional to the size of the enumeration area. This is then followed by a random selection of households within selected enumeration areas. Data collection for the survey is done through face-to-face interviews using questionnaires which are administered to household heads and selected household individuals including women in the reproductive age (15–49 years) who consent to be interviewed. For the purposes of the current study, the following inclusion criteria; (i) only sub-Saharan African countries, (ii) most recent survey conducted between 2008 and 2017 and (iii) the survey included indicators of children’s nutritional status (i.e. stunting, wasting and Hb concentration) and women’s nutritional status (i.e. BMI and Hb level) were applied in extracting data for the analysis. Based on these criteria, a total sample of 145 020 households with women–child pairs from twenty-three countries was realised (Table 1). The data for the current study were extracted from IPUMS (Integrated Public Use Microdata Series) DHS^(37)^. IPUMS uses a data warehousing approach to extract, transform and load data from numerous nationally representative surveys into a single-view schema to ensure that data sources become compatible. A total of twenty-six sub-Saharan African countries were initially extracted from the IPUMS website for analysis in the current study. Appendix 1 shows the characteristics of households as well as the characteristics of women and children under 5 years that were available for each country. Notably, Hb concentration readings for measuring anaemia were not captured for women in the Angola, Kenya, Nigeria, Zambia and Sudan data sets. Additionally, the Sudan data set had no indicator of nutritional status for women and children, while Angola had no data on nutritional status indicators for women. The final analytical sample therefore includes twenty-three countries with valid data on all variables of interest.

**Table 1:** Distribution of study sample by country, survey year and final sample size for analysis

Country	Year	Sample size
Burkina Faso	2010	6649
Burundi	2016	5505
Cameroon	2011	4748
Congo	2013	7863
Benin	2011	10 996
Ethiopia	2016	8751
Ghana	2014	2561
Guinea	2012	3082
Cote d’Ivoire	2011	2804
Kenya	2014	8607
Lesotho	2014	1195
Madagascar	2008	5096
Malawi	2016	4432
Mali	2012	4695
Mozambique	2011	8648
Namibia	2013	997
Niger	2012	5201
Nigeria	2013	26 465
Rwanda	2014	3282
South Africa	2016	438
Zimbabwe	2015	4426
Tanzania	2015	7794
Zambia	2013	10 785
Total		145 020

### Measurement of variables

#### Outcome variables

The study focused on measuring five categories of household burden of malnutrition as dependent variables. These were undernutrition, overweight/obesity, anaemia (micronutrient deficiency), double burden and triple burden malnutrition. These conditions were measured among women (15–49 years) and children (6–59 months) in the household. Undernutrition among children was measured using height-for-age and weight for height *z*-scores, while overweight/obesity was measured using weight-for-height *z*-scores. Undernutrition and overweight/obesity among women were measured using BMI. Micronutrient deficiency was assessed using anaemia as an indicator, and this was measured using the Hb concentration of children under 5 years and women (15–49 years) in the household.

Household undernutrition also referred to as undernutrition only or undernourished households is characterised by households with either an undernourished child and or undernourished woman. A child is considered to be undernourished when she/he is either stunted (height-for-age *z*-scores < –2) or wasted (weight-for-height age *z*-scores <– 2)^(38)^. An undernourished woman is considered as one with a BMI <18·5 kg/m^2^. An overweight/obese household is characterised as a household having an overweight child and or an overweight/obese woman. An overweight child is one with a weight-for-height *z*-score > 2, while an overweight/obese woman is one with a BMI > 24·9 kg/m^2(39)^. Anaemic households are households with either an anaemic child or anaemic woman or both. Children with Hb concentration of ≤ 110–119 g/l and women with Hb concentration of ≤ 100–109 g/l were considered to be anaemic^(39)^.

Household-level double burden and triple burden malnutrition were based on the existence of a combination of the single conditions in the household. A household with DBM was considered as one having the co-occurrence of undernutrition and overweight/obesity in a child and or woman^(1)^ as defined above. Households with TBM were those with the co-occurrence of undernutrition, overweight/obesity and anaemia^(29)^. For example, a household is classified as a triple burden malnutrition household if the there is an undernourished child who is also anaemic with an overweight/obese woman in the same household. Similarly, a household with an undernourished woman who is also anaemic and an overweight child is classified as triple burden malnutrition household. The classification of household DBM and TBM is thus driven primarily by the combination of the nutritional status of the children and women in the household.

#### Independent variables

The current study sought to examine household factors that influence household burden of malnutrition while controlling for the individual characteristics of women and children in the household. These household and individual factors therefore constitute the independent variables for the study. These include the age and sex of the household head, total number of household members (household size), household access to improved water and sanitation (toilet facilities) and the wealth quintile of the household. The location of the household was considered as whether households were in a rural or urban area. The available individual characteristics include the age of children (under 5 years) and women (15–49 years) in the household.

### Statistical analysis

The characteristics of the study sample including the characteristics of the households and the women and children in the households were described using means and percentages. The various categories of household burden of malnutrition were categorised as binary outcomes, that is, households with an undernourished child and/or woman were classified as 1 and 0 otherwise, households with an overweight/obese child and/or woman are represented as 1 and 0 otherwise, households with either an anaemic child and or anaemic woman were represented as 1 and 0 otherwise. Similarly, households that qualify as having a double burden of malnutrition were assigned a score of 1 and 0 otherwise. The same scale was used for households that had triple burden malnutrition. Per these categorisation and given that the various types of household burden of malnutrition were categorised as binary outcomes, the analysis technique employed to explore the correlates of the various malnutrition outcomes was a binary logistic regression. All statistical analyses were performed using the Stata statistical software package version 14·2 (2017; StataCorp). Statistical significance was set at the 5 % α-level (*P* < 0·05).

## Results

### Characteristics of the study sample

The households in the study had about seven members on average, and the average age of the head of the household was about 39 years (Table 2). The heads of the households were mostly males (84·4 %), and only about 6 % of the household heads had higher than secondary level of education. In terms of conditions in the household, a little more than half of the households had improved sources of drinking water, while about two-fifths had improved toilet facilities. Generally, the proportion of households belonging to the various wealth quintiles decreased as wealth quintile increased. Specifically, about a quarter (24·5 %) of the households belonged to the poorest quintile compared with about 16 % who belonged to the richest quintile. In terms of location of residence, about seven out of ten of the households were in rural areas. Children under 5 in the households were about 2 years and 4 month old on average and a little more than half were between 24 and 59 months old. Male children constituted a slightly higher proportion (50·5 %) compared with female children (49·5 %). Women in the reproductive age in the households were about 29 years old on average with those aged 45–49 years constituting the smallest proportion (2·2 %). Also, nearly all the women of reproductive age in the households were married or cohabiting (95·7 %) and about two-fifths (42·5 %) have not had any formal education, while about one-third had attained primary level of education (Table 2).

**Table 2:** Characteristics of study population

Variables	Mean ± sd/percentage (%)	Number
Household characteristics		
Household size (average)		145 020
Mean	6·75	
sd	0·01	
Age of household head (average)		145 020
Mean	38·82	
sd	0·03	
Sex of household head		
Male	84·4	122 318
Female	15·6	22 702
Level of education attained by household head		
No formal education	34·9	50 593
Primary	32·7	47 379
Secondary	26·1	37 811
Tertiary/higher	6·4	9237
Main source of drinking water		
Improved	55·7	80 748
Non-improved	44·3	64 272
Type of toilet facility		
Unimproved	59·2	85 904
Improved	40·8	59 116
Wealth quintile		
Poorest	24·5	35 555
Poorer	21·4	30 994
Middle	19·6	28 465
Richer	18·3	26 539
Richest	16·2	23 467
Location of household residence		
Urban	28·5	41 392
Rural	71·5	103 628
Characteristics of children		
Age continuous (average)		145 020
Mean	28·60	
sd	0·05	
Age (in months)		
0–5 months	10·6	15 394
6–11 months	10·9	15 730
12–23 months	20·6	29 842
24–60 months	57·9	84 072
Sex		
Male	50·5	73 219
Female	49·5	71 801
Characteristics of women		
Age (average)		145 020
Mean	29·4	
sd	0·02	
Age (5-year groups)		
15–19	4·6	6607
20–24	20·6	29 881
25–29	28·2	40 851
30–34	22·3	32 288
35–39	15·1	21 924
40–44	7·1	10 339
45–49	2·2	3130
Marital status		
Single	4·3	6195
Married	95·7	138 825
Highest level of education attained		
No formal education	42·7	61 848
Primary	34·7	50 345
Secondary	19·7	28 516
Tertiary/Higher	2·9	4311
Total	100·00	145 020

### Prevalence of malnutrition among women of reproductive age and under children 5

The results in Table 3 show that a little more than one-third (37·2 %) of the children under 5 involved in the study were stunted and about one in ten (9·9 %) were wasted, while about 5 % were overweight/obese and about two-thirds (63·4 %) were anaemic. Additionally, about eight out of ten of the children under five were undernourished and anaemic, while 2·1 % were overweight and anaemic. Considering women in the reproductive age, one out of ten were underweight, while almost seven in ten were of normal weight and all together, about one in five were either overweight (15·7 %) or obese (5·6 %). Again, about every four out of ten of the women in the households involved in the study were anaemic. Furthermore, about 7 % of the women were overweight and anaemic, while about 5 % were underweight and anaemic (Table 3).

**Table 3:** Prevalence of malnutrition in household

Household population-group	Malnutrition status	Percentage (%)	95 % CI	Number
Children	Stunting			
	Normal	62·8	62·5, 63·1	83 193
	Stunted	37·2	36·9, 37·5	49 301
	Wasting			
	Normal	90·1	90·0, 90·3	115 484
	Wasting	9·9	9·7, 10·0	12 626
	Overweight/obesity			
	Normal weight	95·1	95·0, 95·2	121 843
	Overweight	4·9	4·8, 5·0	6267
	Anaemia			
	Not anaemic	36·6	36·3, 37·0	27 477
	Anaemic	63·4	63·0, 63·7	47 504
	Combined conditions			
	Undernutrition (stunted or wasted) + anaemic	77·9	77·6, 78·2	52 924
	Overweight + anaemic	2·1	2·0, 2·2	1433
Women	BMI			
	Underweight	9·9	9·7, 10·0	14 323
	Normal weight	68·8	68·6, 69·1	99 798
	Overweight	15·7	15·5, 15·9	22 753
	Obese	5·6	5·5, 5·7	8146
	Anaemia			
	Not anaemic	58·7	58·4, 59·0	53 069
	Anaemic	41·3	41·0, 41·6	37 333
	Combined conditions			
	Overweight + anaemic	6·7	6·6, 6·9	6084
	Underweight + anaemic	4·8	4·7, 4·9	4334
Household (Women + Children)				
	Undernutrition (Women + Children)	5·1	5·0, 5·2	6490
	Overweight/obesity (Women + Children)	1·5	1·4, 1·6	1933
	Anaemia (Women + Children)	29·4	29·1, 29·7	21 927

### Household burden of malnutrition

**Fig. 1:**
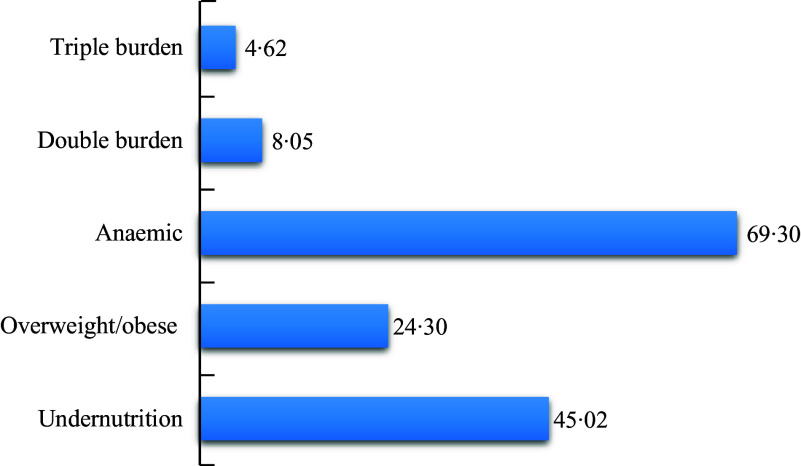
Percentage distribution of household burden of malnutrition

The most common condition of malnutrition at the household level was anaemia which is characterised by the presence of either a child under 5 who is anaemic or a woman (15–49 years) who is anaemic or both. The results in Fig. 1 show that nearly seven out of ten households involved in the study had an anaemic child and or woman. The next most common condition of household malnutrition was undernutrition which is characterised by the presence of either an underweight, stunted or wasted child under 5 or an underweight woman or both. The results show that about one in two (45 %) of the households were undernourished households. Overweight/obese households which are characterised by a household having either an overweight/obese child under 5 or an overweight/obese woman or both was observed to be present in about one in four of the households. About 8 % of the households were dual or double burden households, while about 5 % were triple burden households. Additionally, about 5 % of the households had an undernourished child and an undernourished woman, while about 2 % had an overweight/obese woman and an overweight/obese child. However, nearly a third (29·4 %) of the households had an anaemic child and an anaemic woman in the same household (Table 3).

The country-level results presented in Table 4 show wide variations in the burden of malnutrition across SSA. Anaemia is the most common burden of malnutrition, and the condition is highest in Burkina Faso (88·19 %), Mali (84·53 %), Cote d’Ivoire (82·19 %) and Guinea (81·86 %) and lowest in Rwanda (42·60 %). Undernutrition was highest in Burundi (60·96 %), Madagascar (54·98 %) and Ethiopia (54·36 %) and lowest in Ghana (26·55 %). Overweight/obesity was highest in South Africa where about seven out of every ten households had an overweight/obese woman and or child. The proportion of overweight/obese households observed in South Africa was about three times higher the global SSA figure of 24·14 %. Lesotho recorded the next highest proportion of households with an overweight/obese woman and or child (46·69 %) and again this was about twice the global SSA figure. Madagascar recorded the lowest proportion (5·46 %) of households with overweight/obese women and or children. In general, double and triple burden malnutrition was less common compared with the single conditions. DBM and TBM were highest is South Africa (17·12 %), Benin (13·99 %) and Lesotho (13·81 %) and lowest in Madagascar (1·71 %). Similarly, TBM was highest in South Africa (10·49 %) and Lesotho (8·66 %) and lowest again in Madagascar (1·09 %).

**Table 4:** Percentage distribution of household burden of malnutrition by country

	Household burden of malnutrition(%)				
Country	Undernutrition	Overweight/obesity	Anaemia	Double burden	Triple burden
Burkina Faso	49·47	10·75	88·19	3·74	3·35
Burundi	60·96	10·61	68·29	3·72	2·19
Cameroon	37·97	34·04	70·16	8·80	6·11
Democratic Republic of Congo	53·81	16·86	70·34	7·33	5·01
Benin	39·26	33·14	68·23	13·99	8·51
Ethiopia	54·36	11·10	65·26	3·18	2·04
Ghana	26·55	37·02	74·75	5·78	4·55
Guinea	41·69	20·08	81·86	5·68	4·59
Cote d’Ivoire	35·52	23·32	82·15	6·03	4·68
Kenya	37·63	28·57	–	6·56	–
Lesotho	37·57	46·69	57·78	13·81	8·66
Madagascar	54·98	5·46	62·16	1·71	1·09
Malawi	39·17	24·19	66·69	7·69	4·89
Mali	46·84	19·72	84·53	6·69	5·61
Mozambique	44·55	21·66	64·71	8·37	5·20
Namibia	30·29	36·91	51·41	7·02	4·43
Niger	52·11	22·01	78·20	8·56	6·81
Nigeria	47·22	28·03	–	10·10	–
Rwanda	40·37	30·41	42·60	9·72	4·30
South Africa	24·20	74·66	56·27	17·12	10·49
Zimbabwe	29·17	41·82	45·73	9·60	4·64
Tanzania	40·22	28·42	71·07	8·61	5·98
Zambia	45·74	24·75	–	9·61	–
Total	45·02	24·14	69·30	8·02	4·60

### Household factors as predictors of household burden of malnutrition

The results presented in Table 5 show that the older the head of the household, the lower the chances of the household having a woman and or a child who is undernourished but the higher the likelihood of the household having an overweight/obese woman and or child. Also, the chances of a household having an anaemic child and or woman was about 2 % higher for each additional increase in the age of the head of the household. The age of the household head was, however, not significantly associated with the household experiencing double or triple burden of malnutrition. The sex of the household head on the other hand did not show a significant association with any of the conditions of household burden of malnutrition. Household size on the other hand was associated with the single conditions of household burden of malnutrition. Increasing household size was associated with a higher likelihood of undernutrition and anaemia but a lower likelihood of overweight/obesity at the household level. The results further reveal that unimproved source of drinking water was associated with a lower likelihood of a household being an anaemic or triple burden household. The state of sanitation in the household, that is, whether the household had an improved or unimproved toilet facility was significantly associated with some conditions of household burden of malnutrition. Specifically, households that have improved toilet facilities were more likely to have a child or adult woman who is overweight/obese but less likely to be undernourished. Households with improved toilet facilities also had a higher chance of being double and triple burden malnutrition households.

**Table 5:** Results of binary logistic regression analysis showing the odds ratios of the association between household characteristics and household burden of malnutrition

	Odds ratios									
Variables	Undernutrition (*n* 145 020)	95 % CI	Overweight/obesity (*n* 145 020)	95 % CI	Anaemia (*n* 90 783)	95 % CI	Double burden (*n* 145 020)	95 % CI	Triple Burden (*n* 90 783)	95 % CI
Age of household head	0·997	0·996,0·999***	1·006	1·005,1·008***	1·002	1·000,1·003*	1·002	1·000,1·004	1·003	0·999,1·006
Sex of household head [Male]										
Female	0·970	0·932,1·010	0·985	0·940,1·032	0·988	0·940,1·037	0·931	0·867,1·000	0·965	0·870,1·070
Household size	1·013	1·009,1·018**	0·980	0·974,0·986***	1·028	1·021,1·035***	0·996	0·988,1·004	0·995	0·983,1·009
Household drinking water [Improved]										
Unimproved	0·980	0·953,1·008	0·999	0·996,1·033	0·960	0·925,0·996*	0·954	0·908,1·002	0·909	0·841,0·982*
Toilet facility/sanitation [Unimproved]										
Improved	0·962	0·933,0·993*	1·193	1·150,1·238***	1·004	0·963,1·046	1·153	1·092,1·217***	1·179	1·079,1·287***
Wealth status [Poorest]										
Poorer	0·829	0·797,0·862***	1·423	1·346,1·504***	0·811	0·768,0·856***	1·236	1·145,1·333***	1·103	0·980,1·242
Middle	0·720	0.691,0.750***	1·747	1·652,1·849***	0·767	0·726,0·812***	1·298	1·199,1·404***	1·113	0·988,1·254
Richer	0·604	0·577,0·633***	2·375	2·238,2·520***	0·679	0·640,0·721***	1·409	1·294,1·535***	1·224	1·078,1·389**
Richest	0·449	0·423,0·476***	3·788	3·531,4·063***	0·562	0·522,0·605***	1·507	1·358,1·673***	1·171	0·997,1·376
Location [Urban]										
Rural	1·055	1·016,1·096**	0·838	0·804,0·874***	0·935	0·887,0·986*	0·934	0·887,0·995*	0·975	0·873,1·089
Children (under five)										
Age of child in months [0–5]										
6–11 months	1·453	1·369,1·543***	0·743	0·693,0·796***	5·196	4·808,5·616***	0·838	0·755,0·931**	1·350	1·123,1·622**
12–23 months	2·319	2·199,2·445***	0·692	0·652,0·735***	4·658	4·365,4·970***	0·944	0·863,1·032	1·616	1·376,1·897***
24–60 months	2·295	2·129,2·342***	0·793	0·752,0·836***	2·809	2·658,2·968***	1·019	0·942,1·103	1·631	1·406,1·891***
Women’s Characteristics										
Age [15–19]										
20–24	0·833	0·777,0·892***	1·406	1·266,1·561***	0·964	0·881,1·054	1·048	0·916,1·199	0·856	0·698,1·050
25–29	0·727	0·679,0·778***	1·993	1·799,2·207***	0·878	0·804,0·960**	1·186	1·040,1·352*	1·132	0·929,1·378
30–34	0·710	0·663,0·762***	2·675	2·412,2·965***	0·824	0·752,0·902***	1·442	1·262,1·647***	1·155	0·945,1·411
35–39	0·704	0·655,0·758***	3·209	2·886,3·567***	0·826	0·751,0·909***	1·566	1·366,1·795***	1·257	1·023,1·546*
40–44	0·667	0·615,0·724***	3·312	2·955,3·712***	0·773	0·694,0·861***	1·609	1·385,1·869***	1·443	1·155,1·803**
45–49	0·684	0·613,0·764***	3·385	2·935,3·903***	0·796	0·686,0·925**	1·786	1·479,2·157***	1·385	1·0 341 857*
Marital status [Single]										
Married/Cohabiting	0·929	0·856,0·999	1·276	1·167,1·397***	1·041	0·946,1·146	1·094	0·959,1·247	1·063	0·875,1·292
Level of educational attainment [No education]										
Primary	0·759	0·735,0·784***	1·219	1·171,1·269***	0·811	0·776,0·847***	0·979	0·923,1·037	1·089	0·990,1·199
Secondary	0·583	0·558,0·608***	1·526	1·451,1·604***	0·751	0·708,0·797***	0·951	0·881,1·026	1·029	0·905,1·169
Tertiary/Higher	0·379	0·341,0·420***	2·150	1·962,2·355***	0·549	0·478,0·630***	0·735	0·629,0·860***	0·545	0·374,0·793**
Year of survey [2008]										
2010	0·770	0·703,0·844***	2·045	1·717,2·436***	3·797	3·388,4·254***	1·936	1·437,2·608***	2·789	1·958,3·972***
2011	0·574	0·533,0·619***	7·562	6·494,8·805***	1·109	1·023,1·202*	6·717	5·173,8·722***	5·659	4·109,7·793***
2012	0·772	0·711,0·838***	4·376	3·733,5·129***	2·129	1·941,2·335***	4·108	3·136,5·382***	5·341	3·851,7·409***
2013	0·896	0·834,0·963**	4·906	4·219,5·705***	1·166	1·067,1·275**	5·468	4·217,7·091***	4·341	3·100,6·003***
2014	0·565	0·521,0·613***	6·363	5·443,7·439***	0·693	0·631,0·761***	4·091	3·126,5·353***	4·112	2·931,5·767***
2015	0·620	0·571,0·674***	6·377	5·454,7·456***	1·802	0·992,1·180	4·878	3·730,6·379***	4·563	3·282,6·344***
2016	0·990	0·916,1·069	2·820	2·412,3·297***	1·046	0·964,1·135	2·788	2·131,3·649***	2·555	1·839,3·550***

Reference category *P < 0·05 **P < 0·01 ***P < 0·001.

The results with regard to household wealth status indicate that, on the one hand one, increasing household wealth status was associated with a lower likelihood of a household being undernourished or anaemic, but on the other hand, increasing household wealth was associated with a higher likelihood of the household being overweight/obese. Similarly, the chances of a household being a dual burden malnutrition household increased with increasing household wealth but considering triple burden of malnutrition, only households in the richer wealth category were found to be at increased risk. The location of a household, whether rural or urban, was found to be associated with undernutrition such that rural households were more likely to be undernourished. However, rural households were less likely to be overweight/obese, anaemic or double burden malnutrition households.

Considering the characteristics of reproductive age women and under 5 children in the household, the findings indicate that the chances of a household having an undernourished child and or an underweight woman increases with increasing age of a child under 5, but the chances of a household having an overweight/obese adult woman and or child decreased with increasing age of a child under 5. Additionally, there was a higher likelihood of a household having an anaemic child and or anaemic woman with increasing age of a child under 5; however, the odds was lower at older ages for the child. There was a lower likelihood of a household having a double burden of malnutrition with increasing age of a child under 5 from 6 to 23 months, but statistical significance was only achieved for infants who were aged 6 to 11 months compared with 0–5-month-old infants. Again, the chances of a household having a triple burden of malnutrition generally increased with increasing age of a child under 5 in the household.

Considering the age of women in the reproductive age in the household, the findings indicate that the chances of a household being an undernourished household decreased with increasing age of women from age 20 to 49 years. Similar results were observed for anaemia, but the results were only statistically significant for women aged 25–49 years. The chances of a household being overweight/obese was observed to increase with increasing age of reproductive age women in the household. Again, the chances of a household having a double or triple burden of malnutrition increased with increasing age of reproductive age women from age 25 to 49 and 35 to 49 years, respectively, compared with when a woman in the household is 15–19 years old. The marital status of women in the household only showed a statistically significant association with the chances of a household being overweight/obese. Households with a woman in the reproductive age who was married showed a 26·7 % higher chance of being overweight/obese. Also, increasing level of education among women of reproductive age in the household was associated with a lower likelihood of the household being undernourished or anaemic, but increasing level of education among reproductive age women in the household was associated with increased likelihood of a household being overweight/obese. However, considering the level of education of women in the reproductive age in the household and household double and triple burden of malnutrition, only tertiary level of education (among women in the reproductive age) was observed to be associated with a lower likelihood of a household having a double or triple burden of malnutrition.

Finally, the chances of a household being undernourished decreased in more recent survey years except in 2016, whereas the chances of a household being overweight/obese or anaemic or having a double or triple burden of malnutrition increased in recent survey years.

## Discussion

The current study sought to examine the correlates of different classifications of household burden of malnutrition in SSA. To the best of our knowledge, this is the largest analysis examining household covariates of various conditions of malnutrition; undernutrition, overweight/obesity and anaemia and the co-occurrence of these conditions in the same household, using nationally representative data across sub-Saharan African.

The findings suggest that despite the stereotype image of skeletal children with severe hunger in SSA, the current study like many others, shows that overweight/obesity is also a public health concern in this region^(33,40)^. Our results show that micronutrient deficiency measured as anaemia is the commonest burden of malnutrition among women (15–49 years) and children under 5 in households across SSA. This burden of anaemia corroborates with studies conducted in Arab and Middle East countries^(41,42)^. Reasons for the high prevalence of anaemia in the SSA region are multifaceted. Besides low intake of iron-rich foods, factors such as inadequate hygiene and sanitary conditions are documented as contributors to anaemia in this region. Additionally, conditions such as glucose-6-phosphate dehydrogenase deficiency, haemoglobinopathies endemic diseases such as malaria and sickle cell all contribute significantly to the regions’ anaemia prevalence^(43–45)^. The present study reveals that undernutrition occurs in one in two households, while overweight/obesity was present in about one in every four households in SSA. The results of the current study suggest that the prevalence of single conditions of malnutrition is comparatively higher than double and triple burden of malnutrition. However, the level of DBM as found in the current study is comparable to the level in Nepal (6·60 %)^(46)^. This notwithstanding, an earlier study conducted in Bangladesh, Nepal, Pakistan and Myanmar found the level of household DBM to be slightly lower than that of the present study^(47)^, but the level of TBM as was found in the present study is slightly lower (4·6 %) than what was found in Nepal (7·0 %) by Sunuwar et al.^(46)^.

In addition to the levels of household burden of malnutrition, which was realised, the results indicate that the prevalence of the various forms of household burden of malnutrition and their underlying household influencing factors are different. For example, whereas increasing age of the household head was associated with a lower likelihood of the household being undernourished, the same variable was associated with a higher likelihood of the household being overweight/obese and anaemic. But unlike the age of the household head, the sex of the head of the household head was not associated with any of the forms of household burden of malnutrition. This is contrary to a study conducted in Indonesia that reported some nutritional inequality among female-headed households^(48)^. The size of the household on the other hand was associated with a higher likelihood of undernutrition and anaemia as has been found in other studies^(49)^. These findings are plausible because increasing household size affects access to food such as fruits, vegetables^(50)^, meat and fish which tend to be relatively more expensive. The absence or reduction of such foods contributes significantly to iron-deficiency, causing anaemia. Household size was, however, found to be associated with a lower likelihood of the household having either an overweight/obese woman or child or both. Other household characteristics such as the type of toilet facility was found to be associated with the household’s likelihood of having either an overweight/obese woman or child and also double and triple burden of malnutrition in the household. The mechanism of impact of type of toilet facility on the household burden of malnutrition as found in the present study could be operating through wealth or socio-economic status. Households with improved toilet facilities tend to be relatively wealthier compared with households with unimproved toilet facilities, and such wealthier households have been found to have higher burden of overweight/obesity, especially among women, and this could be what is driving the household burden of overweight/obesity, double and triple burden of malnutrition as was observed in the present study.

In terms of household wealth status, the results indicate that while richer households are less likely to be undernourished or anaemic, they are more likely to be overweight/obese and also, have double burden or triple burden malnutrition conditions. Similar findings have been reported in Nepal where mothers from the richest households were more likely to have double or triple burden of malnutrition^(46)^. Some of the plausible reasons for this finding are that while rich households may be more food secure^(34,51)^ and also benefit from a more diverse diet, thus reducing the risk of undernutrition and micronutrient deficiency, rich households are also at a higher risk of overweight/obesity due to consumption of energy-dense foods and lower levels of physical activity. The location of a household, whether rural or urban, was also found to be associated with household burden of malnutrition. Rural households were found to have a lower likelihood of being overweight/obese or anaemic. Contrarily, other studies in some SSA countries, for example Ghana found that rural residence is associated with a high likelihood of micronutrient deficiency^(34)^. Another study conducted in the Gambia did not find significant difference in anaemia among non-pregnant females by residence^(52)^. These results do show the possible variability in the conditions contributing to anaemia in the region.

The present study found that increasing age of children under 5 years was associated with a higher likelihood of household TBM contrary to the findings of Sunuwar and colleagues in Nepal^(46)^, while in Pakistan and Myanmar, Anik and colleagues found that child’s age (24–59 months) was associated with a higher likelihood of the household experiencing a double burden of malnutrition^(47)^. Additionally, the present study found that child’s age was significantly associated with a higher likelihood of the household being undernourished as was found in Sierra Leone before the Ebola outbreak^(53)^. The effect of the age of children on the household burden of malnutrition could be a result of child feeding practices especially during complementary feeding and the weaning period. Poor feeding practices, feeding children with poor quality and or unbalanced diets and unhygienic feeding practices in the complementary feeding/weaning period increase the likelihood of undernutrition and micronutrient deficiency among children, and this contributes to the household burden of malnutrition.

Considering the characteristics of reproductive age women who are members of the household, the findings with regard to their age were as expected and similar findings have been found among women in Nepal^(46)^. Increasing age among women was associated with a higher chance of the household experiencing double and triple burden of malnutrition. The higher chance of overweight and obesity among older women in sub-Saharan Africa^(54)^ is one of the possible drivers of the higher likelihood of household DBM and TBM as was found in the present study. Again, being in a marital union (for women of reproductive age) was found to be associated with a higher chance of the household being overweight/obese. This could also be because of the higher likelihood of married/cohabiting women being overweight/obese compared with their counterparts who are not married or in union. The level of education of women in the household also showed expected results. Higher level of education among women in the household was associated with a lower likelihood of the household being undernourished or anaemic on one hand but a higher likelihood of household overweight/obesity on the other hand. This pattern of results is likely being driven by other mechanisms influenced by education. For example, educated women are more likely to be employed and or gain income and thus they can contribute to meeting the food expenditure needs of their household which lowers the risk of food insufficiency thereby reducing the chances of undernutrition while also ensuring that the household consumes a balanced and diversified diet and thereby reducing the chances of micronutrient deficiency. But on the other hand, households with educated women may also be at risk of having overweight/obese individuals because of frequent consumption of high calorie energy-dense foods such as polished rice, sweetened carbonated drinks and high-fat high sugar snacks.

The findings regarding the year the survey was conducted gives indication of the ongoing nutrition transition in sub-Saharan Africa. The chances of household undernutrition generally decreased in more recent years, whereas the chances of overweight/obesity and anaemia increased in recent years. Again, the chances of double and triple burden of malnutrition were found to increase in more recent years. Changing dietary behaviours from consumption of nutritious staple/traditional diets rich in fibre and complex carbohydrates to the consumption of high calorie, energy-dense, fatty foods and food with simple sugars in recent years could be contributing to the higher likelihood of overweight/obesity and anaemia and the higher risk of double and triple burden of malnutrition in households in SSA. The high prevalence and higher likelihood of anaemia is particularly concerning. Governments of SSA countries and the international community need to pay attention to this as emphasised by Jiwani *et al.* ^(55)^.

In tackling the double and triple burden of malnutrition, the WHO suggests ‘Double-duty’ actions^(56)^. These can be achieved through integrated initiatives, policies and programmes involving different sectors such as the various health and agricultural sectors in countries. Thus, working within ‘institutional silos’ should be avoided as much as possible when addressing the double and triple burden of malnutrition.

The findings of the current study reveal some important findings about the dynamics between household factors and household burden of malnutrition. The current study is one of the few studies that uses nationally representative data across SSA to investigate correlates of household burden of malnutrition from the perspective of influencing household factors. Other studies have mostly investigated individual-level risk factors while controlling for some household characteristics. The current study is also among the few studies that attempt to investigate the prevalence and influencing factors of double and triple burden of malnutrition at the household level across SSA. The study is, however, challenged by some limitations. For example, it would have been helpful to include dietary diversity at the household level in the analysis but not all countries collected data on this indicator; therefore, household dietary diversity could not be included in the analysis. Including this variable in the analysis if it was available may have given some more insights into the findings of the present study. Another limitation with the current study is the use of anaemia as the only indicator of micronutrient deficiency. It will be helpful for future research to measure other indicators of micronutrient deficiency if such data are available. Additionally, the use of IPUMS data has some limitations due to the differing content of questionnaires used in different countries and the suppression of low-level geographic detail. The latter is, however, done to protect confidentiality of respondents. Additionally, although the DHS to a large extent has consistent variables that are collected across the participating countries, there are some variables that may be missing or collected differently. This presents a situation where the sample (number of respondent) may vary during pooled data analysis as was the case in the present study. These challenges notwithstanding, the current study provides insights into the prevalence and household factors that influence the burden of malnutrition at the household level in sub-Saharan Africa.

## Conclusion

The findings of the current study reveal a high prevalence of various forms of household burden of malnutrition particularly the single burden of anaemia, undernutrition and overweight/obesity. The findings further indicate that undernutrition and now overweight/obesity are not the only forms of malnutrition that affect households in SSA. Anaemia is indeed a persistent problem that needs urgent policy attention. Although rapid economic development and urbanisation in the SSA region has created favourable conditions for overweight/obesity, the persistence of poverty and poor access to resources continue to drive the prevalence of anaemia and undernutrition, and the co-existence of extreme spectrums of malnutrition, i.e. double and triple burden of malnutrition in the same household. Though individuals and/or households may respond differently to similar diet and lifestyles, diet quality is a major factor influencing single, double and triple burden of malnutrition^(57)^. Efficiently managing household double and triple burden of malnutrition will require a life-course approach as suggested by Darnton-Hill *et al*.^(58)^. This is important because undernutrition among children could result in overweight/obesity in later adult years, and undernourished women are likely to give birth to underweight children who are at risk of becoming overweight/obese in later years. Unfortunately, knowledge of the current scenario of DBM and TBM has not necessarily translated in concrete policy action in some countries. For example, even though the ‘Roadmap for Nutrition in South Africa 2013–2017’ and Ghana’s ‘National Nutrition Policy For Ghana 2013–2017’^(59,60)^ both acknowledge the presence of both under and overweight/obesity, no clear recommendation is provided towards curbing their co-occurrence in same households. It thus goes without saying that nutrition programming interventions need to address both undernutrition which is dominated by childhood stunting and increasing overweight/obesity among both children and adults and the co-existence of all these conditions in the same household if SSA is to achieve success in combating the burden of malnutrition in the region.

## Supplementary material

For supplementary material accompanying this paper visit https://doi.org/10.1017/S1368980021001750. click here to view supplementary material
